# Pre-Erythrocytic Vaccines against Malaria

**DOI:** 10.3390/vaccines8030400

**Published:** 2020-07-21

**Authors:** Camila Marques-da-Silva, Kristen Peissig, Samarchith P. Kurup

**Affiliations:** 1 Center for Tropical and Emerging Global Diseases, The University of Georgia, Athens, GA 30602, USA; camilasilva@uga.edu (C.M.-d.-S.); kpeissig@uga.edu (K.P.); 2 Department of Cellular Biology, The University of Georgia, Athens, GA 30602, USA

**Keywords:** malaria, pre-erythrocytic, vaccines, immunization, radiation attenuated, Sporozoites, GAPs, RAS

## Abstract

Malaria, caused by the protozoan *Plasmodium,* is a devastating disease with over 200 million new cases reported globally every year. Although immunization is arguably the best strategy to eliminate malaria, despite decades of research in this area we do not have an effective, clinically approved antimalarial vaccine. The current impetus in the field is to develop vaccines directed at the pre-erythrocytic developmental stages of *Plasmodium*, utilizing novel vaccination platforms. We here review the most promising pre-erythrocytic stage antimalarial vaccine candidates.

## 1. Introduction

Malaria is a mosquito-borne infectious disease caused by the eukaryotic pathogen, *Plasmodium. Plasmodium* has a complex life cycle, with distinct, antigenically discrete developmental stages in both vertebrate and invertebrate hosts. According to the World Health Organization (WHO), in 2018 an estimated 228 million new cases of malaria were reported globally, most of which were distributed among just nineteen countries in sub-Saharan Africa and the Indian subcontinent [1]. Exposure to malaria infections in pregnant women led to the delivery of about 872,000 children with low birthweight, indicating that malaria has broad economic and social impacts well beyond the clinical disease itself. Adult residents in malaria-endemic areas get repeatedly infected by *Plasmodium* and are known to develop naturally acquired immunity with age that prevent them from being clinically symptomatic [2]. Since these individuals do not typically receive antimalarial therapy nor develop sterilizing immunity from repeated infection [3], they are effective reservoirs of the *Plasmodium* parasites in the blood facilitating further transmission of the disease [4,5,6]. Hence, it is widely believed that elimination of malaria will not be achieved without safe, effective and affordable vaccines that offer protection from the different *Plasmodium* species causing malaria in humans.

**Figure 1:**
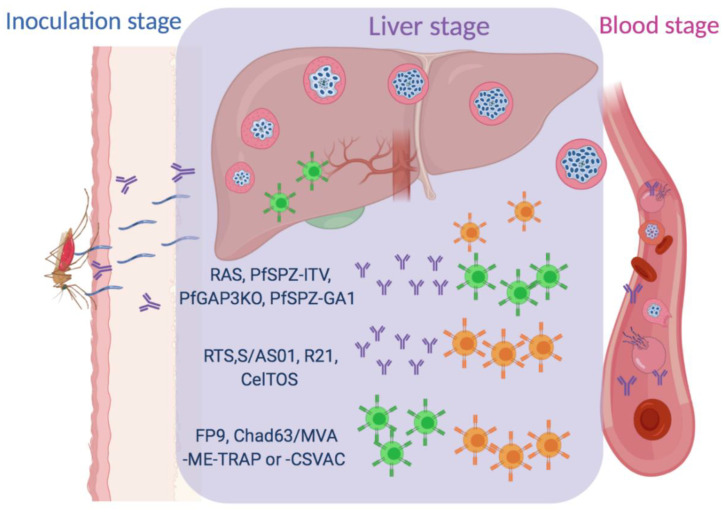
*Plasmodium* parasites are transmitted by infected *Anopheles* mosquitos when they draw a blood-meal from the vertebrate host, and concurrently inoculate the sporozoite stage of the parasite into the skin (inoculation stage). The motile sporozoites exit the dermis and enter the blood stream, travel to the liver and establish an infection in the hepatocytes (liver-stage). The sporozoites undergo asexual replication in the hepatocytes before budding off as hepatocyte plasma membrane-bound merosomes containing infective merozoites into the bloodstream, concluding the pre-erythocytic stage of malaria in the vertebrate hosts. Merosome rupture in the blood vessels to release the merozoites that invade red blood cells, to initiate the blood stage of malaria. Effective pre-erythrocytic-stage vaccines against malaria prevent the invasion and development of *Plasmodium* in the liver. While the RTS, S/AS01, R21 and CelTOS vaccines rely on the antibody and CD4 (orange) T cell responses generated against the constituent *Plasmodium* subunit antigens, the radiation-attenuated sporozoites (RAS), *Pf*SPZ-ITV, *Pf*GAP3KO and the *Pf*SPZ-GA1 vaccines generate protective antibody, CD4 and CD8 (green) T cell responses against multiple *Plasmodium* antigens. The FP9, ChAd63/MVA-ME-TRAP or -CSVAC vaccines rely on the activation of both CD8 and CD4 T cell responses.

In humans, *Plasmodium* sporozoites are delivered into the skin by the female *Anopheles* mosquitos during a blood meal. Subsequently, some of the motile sporozoites leave the bite site and travel through the blood-stream to find, invade and undergo development in the hepatocytes. The developmental progression of the *Plasmodium* parasites from the sporozoite stage inoculated in the skin to the completion of its development in the hepatocytes constitutes the asymptomatic, ‘pre-erythrocytic stage’ of malaria in its vertebrate host (Figure 1). *Plasmodium* development in the hepatocytes (liver-stage) is associated with significant changes in its gene expression and morphology. This process generates tens of thousands of merozoites that are released into the blood from the infected hepatocytes (in vesicles called merosomes), which infect red blood cells and start the symptomatic and highly pathogenic blood-stage of malaria. Preventing the progression of the malaria parasites from the liver-stage to its blood-stage not only prevents the onset of clinical disease and morbidity but also stems transmission. Hence, the development of vaccines targeting the pre-erythrocytic stages of malaria has been a priority in the field for the past few decades.

It is known that the replication of *Plasmodium* parasites in the liver is limited through the coordination of innate and adaptive immune responses [7,8]. Antimalarial vaccines can be broadly classified into three groups—pre-erythrocytic stage vaccines (PEVs), blood-stage vaccines (BSVs) and transmission-blocking vaccines (TBVs). In the PEVs and the BSVs, the hosts are protected by the *Plasmodium*-specific adaptive immune responses that prevent the progression of the infection. The TBVs, however, preclude transmission of malaria by generating neutralizing antibodies against the ookinete antigens in the hosts, which get taken up by the mosquitos along with the blood-meal [9]. PEVs have been a promising immunization strategy against malaria in human trials [10]. These vaccines prevent the onset of clinical disease by blocking the progression of *Plasmodium* to the blood-stage by priming protective adaptive immune responses against the *Plasmodium* sporozoite- or merozoite-stage antigens [7,11]. 

Malaria, like other eukaryotic pathogen infections, presents major challenges when it comes to vaccinations. Our incomplete understanding of the basic immunology of malaria, a relative lack of knowledge of the genetic variability of *Plasmodium* or the intricacies of its infection biology have all been major hurdles to our progress towards an effective vaccine against malaria. An ideal antimalarial vaccine would be safe and offer long-lasting *Plasmodium*-specific adaptive immune responses that are protective against the different *Plasmodium* species in humans. Other practical considerations like the potential for mass production, suitability of the administration route, vaccination schedule, dosage requirements, stability, shelf-life, cost-efficiency etc., are also important, given the target population for antimalarial vaccines live in the poorest parts of the world. Even though research over the years has made significant advances in the area of human malaria vaccines, we are yet to develop an antimalarial vaccine that is broadly protective and practical. We here review the most promising vaccination approaches targeting the pre-erythrocytic stage of malaria currently available or under development, along with the key studies that support them. 

## 2. Subunit Vaccines

Antigen subunit vaccines are designed to prime protective immune responses against a limited number of immunodominant epitopes in a pathogen. The following are the key subunit vaccination approaches pertinent to human malaria infection.

### 2.1. RTS, S/AS01 Vaccine

Although the underlying mechanisms behind eliciting immune responses to the pre-erythrocytic stages of malaria are not fully understood, it is known that protection from new infections can be induced by vaccines that generate strong humoral and cellular immune responses against the sporozoite stage of *Plasmodium* in the mammalian host. While the sporozoite-specific antibodies generated in the immunized host preclude sporozoite invasion of the hepatocytes [12], sufficient numbers of sporozoite antigen-specific T cells help eliminate the *Plasmodium*-infected hepatocytes that present these epitopes [7]. The Circumsporozoite Protein (CSP), that forms an integral part of the surface-coat of the sporozoites, is vital in the interactions between the sporozoite and the host hepatocytes [13,14,15]. This has made the CSP a potential target antigen in pre-erythrocytic-stage antimalarial vaccines. 

Thus, it is not surprising that one of the most prominent antimalarial subunit vaccines is the CSP-based RTS, S/AS01 recombinant vaccine (*Mosquirix*). The 58 kDa CSP protein contains 37–49 repeats of the NANP (N, asparagine; A, alanine; P, proline) amino acids and a smaller number of valine and aspartic acid residues [16]. The CSP C-terminal region contains T cell epitopes recognized by multiple Human Leukocyte Antigens DR isotype (HLA-DR) molecules [17,18]. The RTS, S/AS01 vaccine is composed of the *P. falciparum* (NF54 strain) CSP central repeat region with the NANP amino acid repeat sequence (R) with a B-cell receptor epitope and immunodominant CD4 and CD8 T cell epitopes (T) fused to hepatitis B surface antigens (HBsAg, the S antigen). In addition to this, three times more ‘free’ HBsAg antigen (S) is made available in the vaccine formulation to facilitate the self-assembly of the immunogen into virus-like particles [19]. Protection against malaria through RTS, S/AS01 vaccines is through the induction of high levels of both anti-CSP antibodies and CD4 T cells expressing IL-2, TNF, IFN- and the co-stimulatory marker CD40L [20,21]. The humoral and cellular immune responses generated by the RTS, S/AS01 vaccine were shown to offer immunity to mosquito bite challenge infections in human trials [22,23]. The RTS, S/AS01 vaccination also generated protective immune responses against Hepatitis B, in addition to against malaria [24].

Early formulations of the RTS, S moiety using alum or MPL (3-O-desacyl-4’-monophosphoryl lipid A) as an adjuvant did not induce a robust immune response. None of the six volunteers developed protective immunity with alum alone while two out of eight volunteers were protected with alum-MPL [20,25]. Subsequently, two similar adjuvant formulations made up of MPL and saponin (QS21) were developed for the RTS, S vaccine—AS01, a liposome formulation, and AS02, a squalene-based emulsion formulation. These liposomal adjuvants were efficient at generating a TLR4-mediated cytokine response as well as in inducing costimulatory molecules in the antigen-presenting cells [26,27]. RTS, S in combination with AS01 (RTS, S/AS01) generated stronger CSP-specific humoral and cellular immune responses [28,29], offered protection from experimental *Plasmodium* sporozoite challenges and was the first malaria vaccine to enter Phase III clinical trials [30]. It was shown that 18 months after the third booster dose, RTS, S/AS01 prevented 829 cases of clinical malaria episodes per 1000 children [31]. However, RTS, S/AS01 vaccination did not offer tangible immunity in the infants [31]. In phase III clinical trials in infants and children (ages 1–4), RTS, S/AS01 vaccination offered only 65% or 56% reduction (respectively) in severe malaria [32]. Nevertheless, the RTS, S/AS01 vaccine was approved for clinical use by the European regulatory bodies. In the ‘Expanded Program of Immunization’ in Africa, 8–12 week old children were offered RTS, S/AS01 along with the various childhood vaccines, with the RTS, S/AS01 booster dose delivered at forty-eight months post primary vaccination [33,34]. The co-administration of RTS, S/AS01 with the yellow fever and measles-rubella vaccines and vitamin A supplementation in children helped the program offer it without increasing the number of immunization-clinic visits required [33]. 

The RTS, S/AS01 vaccine, despite its clinical use, is far from being the ideal malaria vaccine. The protection offered is inadequate and wanes over time [31]. The CSP antigen, which the RTS, S vaccine primarily targets, is expressed in the sporozoite stage of *Plasmodium,* which typically invades the liver cells in under half an hour of being inoculated into humans [35,36]. The merozoite stage of the parasite that the sporozoite transforms itself into, and emerges from the hepatocytes 7–10 days later causing clinical malaria, is unrecognized by the RTS, S-induced antibodies or T cell responses. Preventing any sporozoites from reaching the liver in the short window of opportunity available appears a practically difficult task even with strong circulating antibody responses [30]. In addition, waning CSP-specific immune responses from RTS, S/AS01 vaccination is a significant shortcoming. In RTS, S/AS01 infant vaccination programs, worn-off immunity has resulted in rebound or age-shift of infections [37]. Besides that, the RTS, S vaccine has also raised some safety concerns in children [37,38]. The RTS, S vaccine does not possess the CSP N-terminal region in its formulation, although the naturally acquired antibodies to the CSP N-terminal are associated with protection from malaria [39,40]. When longer synthetic peptides containing the entire N- or C-termini of CSP were prepared with suitable adjuvants, stronger, more effective T cell and antibody responses were generated [41,42]. The development of better, more potent adjuvants may also help offer stronger immune responses that are sustained longer. The structural data emerging from CSP-specific antibodies could also provide key insights into improving the CSP-based vaccines [43]. The advent of novel vaccination approaches and platforms may help develop better CSP-based antimalarial vaccines in the future [44,45]. 

### 2.2. R21

R21 is considered the next generation antimalarial subunit vaccine, and is an improved version of the RTS, S/AS01 vaccine. The R21 design incorporates a higher proportion of *Pf*CSP C-terminus bound to HBsAg N-terminus without the three-fold molar excess of HBsAg found in RTS, S/AS01 [20,46]. In this way, more CSP antigen is displayed on the vaccine particle surface, mimicking the higher CSP epitope concentration on the *Plasmodium* sporozoite surface [43]. This enhanced B cell activation led to stronger anti-CSP humoral immune responses [47]. In BALB/c mice, R21 was immunogenic even at very low doses. When administered with the adjuvants Abisco-100 and Matrix-M, R21 elicited protection against the *Pf*CSP-transgenic *P. berghei* sporozoite challenge [48]. A clinical trial using R21 with the AS01 adjuvant was well-tolerated and induced a strong anti-CSP antibody response [30]. R21 is now under evaluation in Phase 1/2a clinical trials [49]. 

### 2.3. Viral-Vectored Vaccines

Another strategy used to formulate an antigen-subunit vaccine for malaria is to utilize viral vectors to deliver the antigens. Homologous or heterologous prime-boost vaccinations using viruses encoding the *Plasmodium* pre-erythrocytic antigens would induce cell-mediated immune responses [48]. One of the earliest attempts at using this approach employed the use of the Fowlpox Virus 9 (FP9) or the attenuated vaccinia virus (Ankara, MVA) as vectors, either independently or in combination [50,51,52] and expressing the insert ME-TRAP. ME is a string of 20 epitopes from the *P. falciparum* pre-erythrocytic stage that are predominantly targeted by the CD8 T cells [53]. ME is fused to the *P. falciparum* (T9/96 strain) pre-erythrocytic-stage protein, thrombospondin-related adhesion protein (TRAP) to make ME-TRAP [37] that targets multiple developmental stages of the *Plasmodium* parasite in the mammalian host [38]. 

Although priming with FP9-ME-TRAP followed by MVA-ME-TRAP booster induced antigen-specific CD4 and CD8 T cell responses, only two out of the five vaccinated subjects were protected [51]. This resulted in the transition to the ChAd63/MVA ME-TRAP vaccine [54,55]. The ChAd63/MVA ME-TRAP vaccine uses the chimpanzee adenovirus 63 (ChAd63) in addition to the MVA and the ME-TRAP antigen. Prime-boost immunization with ChAd63/MVA ME-TRAP generated high levels of effector CD4 and CD8 T cell responses [55] and in phase 1/2a clinical trials elicited immune protection from heterologous sporozoite challenge in 21% and delayed time to blood stage parasitemia in 36% of the immunized volunteers [54]. In phase 2b clinical trials with this vaccination approach, while Kenyan men showed a 67% reduction in risk of malaria infection, no such benefit was observed in Senegalese men [56,57]. This suggested that the ChAd63/MVA ME-TRAP vaccine may not have a reliable, consistent efficacy in the genetically heterogenous malaria-endemic regions. 

Other alternatives to the ME-TRAP antigen have been explored for antimalarial viral-vector vaccines. One of these is CSVAC, which encodes *Pf*CSP with a truncated C-terminal lacking the 14 amino acids corresponding to the CSP GPI anchor moiety [58]. ChAd63/MVA-CSVAC presented good safety profiles but had no significant improvement in immunogenicity. It is, however, not surprising that ME-TRAP is a better antigenic insert for viral-vectored malaria vaccines [56,58] since cell-mediated immunity is the primary protective tool in viral-vectored vaccines. More recently, a viral-vectored malaria vaccine that combined *Pf*TRAP with the highly conserved merozoite proteins, liver-stage antigen 1 (LSA1) and the liver-stage-associated protein 2 (LSAP2), was developed. Nevertheless, neither its immunogenicity nor protective efficacy were significantly impacted with the inclusion of these two proteins [59,60,61].

The relative success of the viral-vectored malaria vaccines is promising due to their ability to induce high levels of T cell responses. Yet, their efficacy in the malaria-endemic regions have been less than ideal. It is possible that inserting more immunogenic *Plasmodium* antigens, as they are identified, into the viral vectors may change this. Additionally, a combination of viral-vectored malaria vaccines with RTS, S/AS01 may also improve the overall vaccine efficacy by the generation of protective CD8 and CD4 T cell responses along with the strong anti-CSP antibodies. 

### 2.4. CelTOS

Besides the CSP and TRAP proteins, the cell-traversal protein for ookinetes and sporozoites (CelTOS) of *Plasmodium* has been considered an attractive target antigen for the development of pre-erythrocytic malaria vaccines. CelTOS is a 25-kDa protein vital for the ookinete traversal in the mosquito midgut, as well as for the sporozoite infection of liver cells in the human host, which also primes a strong immune response [62]. Additionally, the CelTOS protein sequence is highly conserved among the various *Plasmodium* species [63,64]. In both inbred and outbred mice, immunization with recombinant *Pf*CelTOS adjuvanted with Montanide ISA 720 resulted in potent humoral and cellular immune responses that induced cross-reactive immunity against *P. berghei* sporozoite challenge [65]. More recently, it was shown that vaccination with recombinant *Pf*CelTOS when formulated with suitable adjuvants would induce high-affinity antibodies that inhibit *P. falciparum* infection of *Anopheles stephensi* mosquitos [66], suggesting that *Pf*CelTOS may be an effective vaccine to not only prevent clinical infection in the human host but also transmission of the infection to and from mosquitos. Despite the positive results, the CelTOS vaccine will require more refinement before being considered a potential malaria vaccine. 

A key deficiency of subunit vaccines is their inability to generate long-standing immunity. Novel adjuvants and vaccine delivery platforms may help induce long-lived plasma cells to help improve the longevity of subunit vaccine-induced memory responses. This was demonstrated by the partial success of the preclinical study combining the FP9 and the MVA viral vectors with a CSP-based Hepatitis B virus core particle [67]. Another major shortcoming of the subunit vaccines, particularly against complex eukaryotic pathogens with multiple life-cycle stages in a host, is that the immune responses generated against a single or a few epitopes are often not enough to offer effective immunity. Although using multivalent antigenic preparations might negate this shortcoming to some extent, the problem is exacerbated by the inherent genetic diversity of the *Plasmodium* parasites. For instance, the RTS, S/AS01 vaccine efficacy is noticeably lower when challenge infections do not match the 3D7 strain-derived CSP isotype. By combining multiple antigens from different stages of *Plasmodium* development in the mammalian host, targeting highly conserved *Plasmodium* antigens with fewer polymorphisms or using novel adjuvants that induce stronger innate and adaptive immune response, some of the deficiencies of subunit antimalarial vaccines may be overcome at least partially. 

## 3. Whole Sporozoite Vaccines

It has been demonstrated that *P. falciparum* infection initiated by the sporozoites, when arrested in its liver stage of development, provide immunity from *P. falciparum* challenge in humans [68]. *Plasmodium* infection can be prevented from causing clinical malaria by using either chemoprophylactic antimalarial drugs that are specific to blood-stage malaria, or by using attenuated *Plasmodium* (by irradiation or targeted genetic alteration) parasites that prevent its progression beyond the liver. While the chemoprophylactic drugs prevent the progression of *Plasmodium* beyond the first round of blood-stage infection, the attenuated *Plasmodium* do not complete their liver-stage development and fail to enter the blood-stage altogether. 

### 3.1. PfSPZ with Chemoprophylaxis (PfSPZ ITV)

The infection treatment vaccination (ITV) approach in malaria involves infection with wild-type sporozoites (SPZ) under the blood-stage-specific antimalarial drug coverage. Compared to the other whole-sporozoite vaccination approaches, the presence of an abortive blood-stage infection in ITV offers the added advantage of the generation of humoral immune responses against blood-stage malaria antigens. Rodent studies indicated that ITV with chloroquine (CQ) offered protection through the generation of IFN-^+^ CD8 T cell responses [69] directed at the liver-stage of malaria [70]. 

The early human whole-sporozoite vaccination studies began with controlled human malaria infections (CHMI) in conjunction with CQ prophylaxis to prevent blood-stage malaria infection. This immunization method was shown to be protective against homologous strain challenges in 100% of the vaccinated volunteers [71], with some volunteers remaining immune even after 2 years [72]. Protection was shown to be mediated by memory T cells producing IFN-γ, TNF, and IL-2 [71]. However, limited protection was observed following challenge with a heterologous strain of *P. falciparum,* indicating the inadequacy of this approach to protect against the parasite’s genetic diversity [73]. New pharmacological alternatives to chloroquine such as atovaquone/proguanil, azithromycin and pyrimethamine are being developed for CHMI, along with different regimens for immunization [74]. Nevertheless, the risk of not achieving complete parasitological cure with drug treatment, the emergence of drug-resistance [75] and the need for close monitoring of vaccinated subjects with rigorous follow-up, pose major technical and practical hinderances to using this approach as a strategy for mass immunization in endemic areas.

### 3.2. Radiation Attenuated Sporozoites (RAS)

Gamma irradiation of *Plasmodium* sporozoites induces DNA damage in the parasites, leading to the early arrest of its development in the liver. The RAS vaccine contains gamma-irradiated SPZ isolated from the salivary glands of infected mosquitos [76]. Inoculation of RAS leads to abortive infections in the liver that also help prime protective cell and humoral immune responses [77,78]. In the RAS vaccination, protection is mediated by both the humoral immune responses that inhibit the sporozoite motility and invasion of hepatocytes and the T cell-mediated cellular responses directed at the infected hepatocytes [79]. Notably, studies using the mouse model demonstrated that the route of inoculation was also critical in the ability of the RAS vaccines to impart protection. Subcutaneous or intradermal routes of inoculation required 7–10 times more *P. yoelii* RAS to confer protection, suggesting that the IV route of administration is the most efficient to generate T cell responses in animal models [78,80,81,82]. A subsequent study showed that intravenous delivery of *P. falciparum* RAS was crucial to induce high levels of circulating and liver-resident *P. falciparum*-specific CD8 T cells in macaques [83]. Mice immunized with *P. berghei* RAS were also protected from both homologous and heterologous (with *P. vinckei)* [84] species challenges.

The first clinical trials with *P. falciparum* RAS in humans relied on immunization via mosquito bites [74,85]. Although the subcutaneous and intradermal inoculation of the RAS vaccine would more practical, using these resulted in suboptimal immune responses and protection compared to the intravenous route [83]. Intravenous immunization with *P. falciparum* RAS showed that it reduced the risk of malaria infection in malaria-endemic regions, with the protective immunity being associated with robust *P. falciparum* specific CD8 T cell responses [86]. Although the RAS vaccine has been shown to be protective in controlled human trials, one main drawback of RAS is that the parasites are inconsistently, and in some instances incompletely attenuated, both of which can result in ineffective immunity or breakthrough malaria infections [87]. This has led to the push for genetically attenuated sporozoite vaccines where consistent attenuation of sporozoites could be achieved. In addition, utilizing RAS vaccination against malaria face many technical challenges regarding its delivery and administration. RAS vaccines, like other whole-sporozoite vaccines, require the maintenance of a cold-chain during its transport and storage to retain its stability and viability. These are significant challenges in the impoverished malaria-endemic regions. 

### 3.3. Genetically-Attenuated Parasites (GAPs)

Considering the relative success of the whole-sporozoite based vaccines, novel advances in genetic manipulation were used to develop a targeted approach to attenuate *Plasmodium* parasites. By modifying or deleting key essential genes, *Plasmodium* parasites were generated that were incapable of transitioning from its liver-stage to the blood-stage of infection, thereby preventing the onset of clinical malaria. This approach to attenuate *Plasmodium* can be tailored to not affect the viability of *Plasmodium* in its blood-stage and the mosquito vector, infectivity in the mosquitoes, or the transition to the sporozoite stage, to together facilitate generating the transgenic parasites [88]. Targeted genetic alteration of *Plasmodium* also generates a homogenous population of attenuated parasites with a distinct genetic identity. The attenuated status of this unique clonal population also does not rely on external technical variables like the radiation dosage (in RAS) or host drug metabolism (in ITV) [89,90,91]. 

In order to identify the target genes, which when ablated would prevent the progression of the *Plasmodium* parasites beyond its liver stage, a differential gene expression study was performed [88,92] in the liver-stage of malaria [93]. The UIS3 and UIS4 genes, that encode proteins in the parasitophorous vacuole membrane (PVM) of *Plasmodium* liver-stage, were identified as possible targets for gene disruption based on this strategy. In mice, *P. berghei (Pb)* liver-stage development was disrupted in both *uis3* (*Pb uis3*KO) [88] and *uis4* (*Pb uis4*KO) [94] single-knockout parasites and immune protection was conferred upon immunization with either of these parasites; this was dependent on the CD8 T cell responses generated [94]. Nevertheless, breakthrough blood-stage infection was occasionally seen using *Pb uis4*KO and was thus not a viable vaccine candidate. Immunization of mice with double-knockout of *uis3* and *uis4* (*Pb uis3*/*uis4*dKO) prevented any breakthrough parasitemia, while concurrently generating IFN-γ-producing effector and memory CD8 T cell responses that offered immune protection lasting up to 6 months [95]. 

P36, P36p (P52) and B9 were additional candidate genes identified as targets for genetic attenuation, due also to them being highly conserved among the different *Plasmodium* species [96]. All three proteins are members of the 6-Cys protein family and were shown to be essential for the liver-stage development of the *Plasmodium* parasite. In mice, although the *Pb P36*KO infection induced protective immunity, breakthrough parasitemia was observed [97]. Similarly, vaccination with *Pb B9*KO also led to breakthrough infections [98]. However, *P. yoelii (Py) p52*/*p36*dKO*-*vaccinated BALB/c mice exhibited no breakthrough parasitemia and achieved protective immunity from subsequent *P. yoelii* sporozoite challenge [99]. Breakthrough parasitemia was later observed in C57BL/6 mice infected with *Pb p52*/*p36*dKO parasites, suggesting incomplete attenuation of these GAPs [100]. Another gene, the sporozoite asparagine-rich protein 1 (SAP1), observed to be essential for the liver-stage development of *P. yoelii,* was ablated to generate *Py sap1*KO. The *Py sap*1KO parasites exhibited arrest of development in the liver-stage with no breakthrough parasitemia, conferring long-lasting immune protection against wild-type sporozoite infection [101]. 

An ortholog of SAP1 called SLARP was also studied, and *Pb slarp*KO showed similar results to *Py sap1*KO, arresting early in liver-stage development with no breakthrough parasitemia [101]. However, *Py slarp1*KO immunization did not confer long-lasting protective immunity [101]. The deletion of the *FabB/F* gene involved in fatty acid synthesis resulted in *P. yoelii* parasites that arrested late in the liver stage [89]. Immunization with *Py FabB/F*KO elicited a high magnitude of CD8 T cell effector and memory response in both inbred and outbred mice [91]. When compared to the RAS that often arrested early in its liver-stage development, the GAPs that progressed into late-liver-stage engendered higher magnitudes of effector and memory CD8 T cell responses, possibly also directed at a more diverse set of antigens, resulting in better protection from secondary challenges [91]. This finding suggested that late-arresting GAPs may be more reliable as a vaccine candidate, compared to either the early-arresting GAPs or RAS vaccines for malaria. The rodent studies demonstrated that GAP vaccination is protective and mostly safe, pushing the field toward developing GAP vaccines for human malaria [91].

The first clinical trial using GAPs evaluated the safety and immunogenicity of *P. falciparum* lacking the two genes—p52 and p36. The *Pf p52*/*p36*dKO inoculation by mosquito bite was well-tolerated, but one out of the six volunteers develop breakthrough blood-stage malaria infection [102], similar to the observation made in the rodent studies with the *Pb p52*/*p36*dKO parasites [100]. A triple-gene deletion GAP (*Pf*GAP3KO) was subsequently created, deleting *p52*, *p36*, and *sap1* to further reduce the chances of breakthrough parasitemia. Administration of *PfGAP3*KO by mosquito bites in volunteers showed no breakthrough blood-stage *P. falciparum* infection. Considerable anti-CSP antibody titers were generated in all test subjects with significant IFN-γ, IL-2 and TNF cytokine levels detected in the serum [90]. Inspired by the positive results from *Pf*GAP3KO, another GAP vaccine was developed, called the *Pf*SPZ-GA1, lacking B9 and SLARP [98]. Although *Pf*SPZ*-*GA1 offered acceptable safety profiles in Phase I/IIa clinical trials, only three out of the 25 vaccinated volunteers showed protective immunity to mosquito-bite challenge [103].

Given that GAP vaccines are, by design, precisely genetically engineered to prevent the progression of *Plasmodium* beyond its liver-stage of development, they are seen as better pre-erythrocytic malaria vaccines compared to irradiated sporozoites. GAP vaccines are also homogenously attenuated and more broadly immunogenic, making it the safer and better choice for field application [104]. By identifying additional target genes that can be knocked-out to generate GAPs, better vaccine candidates may be generated in the future. A novel approach to generating GAPs is the use of the CRISPR-Cas9 system to attenuate parasites [105,106,107,108]. Although the whole-sporozoite vaccination strategies described above generate protective immune responses, the requirement for intravenous needle delivery of the sporozoites to achieve this is practically challenging in malaria-endemic areas. This caveat also makes the whole-sporozoite vaccinations more difficult in infants and younger children, who constitute the key target age groups for a malaria vaccine [80]. Transgenically expressing strong pathogen-associated molecular patterns (PAMPs) in the GAPs may help enhance the immunogenicity of the RAS-based vaccines, making them more immunogenic and effective when delivered through intravenous or other parenteral routes [109]. The logistics of cryopreservation, maintenance of cold-chain and the need for adequately trained personnel for intravenous delivery of the vaccine are hindrances for the use of any live-attenuated sporozoite based vaccines in mass vaccination campaigns in malaria-endemic areas.

## 4. Conclusions

The field of malaria vaccine development has faced many challenges over the years. The genetic and biological complexities of the *Plasmodium* parasite, presence of multiple, antigenically distinct life cycle stages in the mammalian host, the involvement of mosquito vectors and the prevalence of malaria in the poorest, most populous parts of the world are just a few of these. These have also impeded our ability to understand malaria biology and effectively pursue vaccination strategies to combat this disease. Gaining a better mechanistic understanding of the immune responses mounted against *Plasmodium* infection might help us devise suitable vaccination approaches that effectively prevent the infection. A deeper understanding of *Plasmodium* development in its pre-erythrocytic stage may help unveil novel antigenic targets, amenable to improved vaccination approaches. Together, these can help develop new immunization strategies for the induction of a more effective and functional protective immunity to malaria. Despite the challenges, a great deal of progress has been made in the field of malaria vaccinology. We have made great strides in identifying and testing novel vaccine candidates and resolving the immunological metrics of protection in human and animal models [7,110]. Based on our current understanding of how immune responses help clear *Plasmodium* infection in the liver to prevent the onset of blood-stage malaria, the possibility of developing a clinically applicable, highly effective, conventional subunit vaccine against malaria appears less likely. Although live-attenuated vaccination approaches hold the most promise in terms of inducing protective immune responses at the moment, we have ways to go before these can be safely deployed in the field. We believe that future studies that systematically dissect the immunobiology of liver-stage malaria will help drive the design, development and optimization of effective immunization strategies that will one day help eradicate malaria.
